# Geometric constraints and cognitive inputs jointly shape emergent brain dynamics and topology

**DOI:** 10.64898/2026.08.07.742341

**Published:** 2026-08-07

**Authors:** Ahmad Beyh, Jason Z. Kim, Waheed U. Bajwa, Linden Parkes

## Abstract

How the brain's physical geometry gives rise to its flexible functional repertoire remains a central question in neuroscience. Here, we trained three classes of recurrent neural networks (RNNs) on a working-memory task, forming a graded hierarchy of spatial constraints: Vanilla RNNs (no spatial constraints), Masked RNNs (projection constraints limiting where information enters and leaves the network), and biophysical RNNs (bioRNNs; projection constraints and spatial embedding of the networks' connectivity using the brain's inter-regional Euclidean geometry). We assessed how well each RNN class predicted empirical fMRI activity without exposing them to it during training. Our results showed that bioRNNs were the only networks to successfully predict empirical brain activity and to organize their dynamics into a spatial pattern that recapitulated the brain's principal hierarchy (the sensorimotor–association axis). Additionally, bioRNNs' ability to predict empirical brain activity emerged along a trajectory in which geometry was laid down first, then partly traded back as the task was mastered. Importantly, brain-like topological features emerged in bioRNNs as they increased their task proficiency while maintaining their ability to predict brain activity. Taken together, our results indicate that physical geometry and cognitive inputs play distinct, complementary roles: while geometry constrains the space of possible brain dynamics, cognitive inputs determine which dynamics are expressed. They also situate topology as the scaffold through which the physically embedded brain reconciles wiring costs and computational demands.

## Full Text Availability

The license terms selected by the author(s) for this preprint version do not permit archiving in PMC. The full text is available from the preprint server.

